# ‘Pizza every day – why?’: A survey to evaluate the impact of COVID‐19 guidelines on secondary school food provision in the UK

**DOI:** 10.1111/nbu.12496

**Published:** 2021-06-04

**Authors:** Kelly Rose, Claire O’Malley, Laura Brown, Louisa Jane Ells, Amelia A. Lake

**Affiliations:** ^1^ Centre for Public Health Research School of Health and Life sciences Teesside University Middlesbrough UK; ^2^ Fuse ‐ Centre for Translational Research in Public Health Newcastle upon Tyne UK; ^3^ School of Clinical and Applied Sciences Leeds Beckett University Leeds UK

**Keywords:** adolescent, COVID‐19, nutrition, school food, school policy, young people

## Abstract

The nutritional requirements of adolescence and the reported poor UK eating behaviours of young people are a significant public health concern. Schools are recognised as an effective ‘place’ setting to enable improvement to nutrition outcomes. The COVID‐19 pandemic resulted in UK school closures from March 2020. In re‐opening in September 2020, schools were required to meet guidelines to ensure the minimised impact of COVID‐19 on the population (DfE 2020). We aimed to evaluate the impact of COVID‐19 school guidelines on secondary and post‐16 (16–18 years) food provision. An online survey was posted on 8th October to 1st December 2020, targeted at young people, parents and staff of secondary/post‐16 education establishments in the UK. Two hundred and fifty‐two responses were received, of which 91% reported a change in their school food provision, 77% reported time for lunch was shortened and 44% indicated the provision was perceived as less healthy during September 2020 (post‐lockdown school return) compared with March 2020 (pre‐lockdown). Analyses demonstrated that time, limited choice and healthiness impacted negatively upon young people's school food experience. The COVID‐19 pandemic has presented a huge challenge to the delivery of healthy school food to young people. Therefore, schools require more support in following national food standards and incorporating nutrition education and behaviour change strategies within current guidelines.

## INTRODUCTION

The critical and rapid cognitive, emotional and physical developmental changes experienced during adolescence require a high quality of nutrition (Alberga et al., 2012). However, adolescent eating behaviours in the UK are a cause of significant concern (PHE, 2019). Poor nutrient intake may significantly impact health in the shorter‐ and longer‐term, with malnourished young people more likely to suffer with psychosocial problems and less likely to perform well in school (Kim et al., 2016; Leyva et al., 2020; Story et al., 2009). Young people in the UK tend to consume the lowest number of vegetable and fruit portions, and highest proportions of free sugar in comparison with other age groups (PHE, 2019). Irregular eating patterns, skipping meals and habitually consuming foods high in fat, sugar and salt (HFSS) are also observed as cultural norms within adolescence (Taher et al., 2020).

### Impact of COVID‐19 on young people's eating behaviour

During and post‐COVID‐19 lockdown, young people reported varied experiences of eating behaviours. The absence of routine seemed to impact snacking habits and adverse mental health effects, more so in those from lower income households (Biteback, 2020). Public Health England (PHE, 2020) described the negative health impact of COVID‐19 on dietary and activity habits. Children and young people with overweight/obesity reported a significant increase in using food to manage their emotions, with many showing an awareness and concern for the heightened risk of COVID‐19 due to obesity‐related conditions (PHE, 2020). Indeed, the negative effects reported for children and young people's dietary intake, for example increased skipping of meals and a reduction of fruit and vegetable consumption, combined with a loss of education in school closures, raise concerns that children living in highly deprived communities will be the most disadvantaged in returning to school (Defeyter & Mann, 2020). Recommendations from the *Hungry for Change* report outlined the need for improved food environments (Biteback, 2020). The complexity of the influences involved in adolescent eating behaviours; provide challenges in improving nutrition and reducing obesity levels (Alberga et al., 2012; Jones et al., 2007). The negative health impacts associated with a high consumption of ultra‐processed foods (Pagliai et al., 2020) mean significant changes to environmental structures and access to products high in saturated HFSS are critical to drive positive health change (Jones et al., 2007). Research shows young people prioritise factors including social time, autonomy, cost and convenience, and perceive unhealthy options as low risk to their health (Wills et al., 2018). These views, beliefs and cognitive changes may contribute to the ease of targeting opportunities from HFSS marketing (Murphy et al., 2020). Young people are influenced by their peers, and the perceived social undesirability of the consumption of healthy foods, in contrast to the pleasure associated with HFSS products, is a barrier to improving nutrition outcomes (Wills et al., 2018).

### The school setting in the role of young people's healthy food intake

Poor school food provision is a further barrier to children and young people's healthy food intake (WHO, 2006), and without evaluation of the current UK school food standards established in 2015, it is challenging to assess its effectiveness (DfE, 2019; GNR, 2020; Rose et al., 2019). Schools are recognised as an effective ‘place setting’ to usefully and collaboratively intervene within school and community, to improve nutrition outcomes and reduce health inequalities (NAO, 2020). Incorporating whole systems approaches and multilevel interventions in schools to promote healthy eating has been associated with increased healthy food choices (Townsend & Foster, 2013). Prioritising healthy food provision and nutrition education as a consistent part of the school agenda can have a positive impact on children and young people's wellbeing, learning and academic performance (Rose et al., 2021; WHO, 2006), whilst addressing food insecurity (Defeyter & Mann, 2020).

In September 2020, schools reopened after the lengthy period of closure (March–July 2020). The Government guidance on re‐opening provided information which would support schools in delivering school meals whilst ensuring year groups did not mix, and that each year group would remain in a ‘bubble’ (DfE, 2020). Advice included hygiene protocols, and for schools to consider staggered lunch sittings, or multiple serving areas. It acknowledged the limitations presented by the strict cleaning and social distancing measures, and suggestions were included to simplify lunch menus and reduce choice, recommending that a hot meal be offered when possible (School Food Plan Alliance, 2020; DfE, 2020). The School Food Standards (DfE, 2019) must be adhered to (School Food Plan Alliance, 2020), with the exception of academies established post‐2010 and pre‐2014, which are encouraged to follow the guidance (Rose et al., 2019). This research aimed to investigate the perceived impact of COVID‐19 on school food provision for pupils aged 11–18 years, through an online questionnaire aimed at students, parents, school staff, exploring their experiences of school food choices before and after the first UK lockdown.

## METHODS

A cross‐sectional online survey (Table S1) was developed to explore the views of young people aged 16–18 years, parents of young people (aged 11–18 years) and staff in attendance of secondary or higher education (16–19 years) establishments in the UK, on school food choices following the COVID‐19 school guidelines (DfE, 2020). The STROBE guidance for reporting of observational studies was followed (Von Elm et al., 2014).

Data collection took place between 8th October and 1st December 2020. A survey was developed (hosted on Jisc Online Surveys), and a snowball method was used through social media (Twitter, Facebook, Instagram). The survey was also shared via email (university colleagues, school contacts).

### Data analysis

**FIGURE 1:**
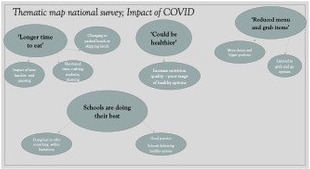
Thematic map of analysis; parents, staff, student's views [Colour figure can be viewed at wileyonlinelibrary.com]

The survey comprised of 11 closed questions to identify the participants’ demographic information (Table 1) and two free text questions. Descriptive analysis was conducted for all quantitative data fields, and thematic analysis was used to analyse all qualitative data arising from the free text fields (Braun & Clarke, 2006). The thematic analysis followed a structured approach (Braun & Clarke, 2006), with codes and themes assessed by researchers, COM and AAL, before reviewing and creating a thematic map of the analysis as shown in Figure 1.

**TABLE 1:** Demographic characteristics of survey respondents

	Parent	Staff	Young person 16–18 years
Respondents, *n* (%)	152 (60%)	61 (24%)	39 (16%)
Geographical location (%)			
North East	88%	46%	73%
North West	5%	10%	6%
East Midlands	1%	14%	8%
West Midlands	0	3%	0
South East/London	5%	17%	14%
South West	0	3%	0
Northern Ireland	0	2%	0
Scotland	1%	3%	0
Wales	0	2%	0
Ethnicity, *n* (%)			
White English/Welsh/Scottish/Northern Irish/British	141 (93%)	51 (84%)	32 (82%)
Peruvian	1 (1%)	0	0
North African/Asian/white English	1 (1%)	0	0
Egyptian and Italian	0	0	1 (3%)
Any other white background	1 (1%)	4 (7%)	0
Arab	1 (1%)	0	0
Black/African/Caribbean/Black British	5 (3%)	0	5 (13%)
Indian	0	3 (5%)	0
Pakistani	1 (1%)	1 (2%)	0
White and Asian	0	1 (2%)	1 (3%)
White and Black African	1 (1%)	0	0
White Gypsy or Irish Traveller	0	1 (2%)	0
Educational establishment, *n* (%)			
Secondary academy	88 (58%)	31 (51%)	6 (15%)
Secondary school	53 (35%)	30 (49%)	9 (23%)
College	7 (5%)	0	17 (44%)
Post‐16	4 (3%)	0	7 (18%)

## RESULTS

**FIGURE 2:**
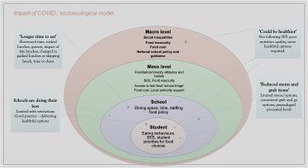
Impact perceptions within a socioecological model [Colour figure can be viewed at wileyonlinelibrary.com]

There were 252 respondents to the survey, the demographics of which are presented in Table 1. The majority of responses were from parents (*n* = 152, 60%), followed by staff (*n* = 61, 24%) and young people (aged 16–18 years; *n* = 39, 16%). Although responses were gained from a range of ethnicities across the UK, the majority of responses were received from white British citizens, largely residing in the North East of England. Half of the survey respondents specified secondary academy as their education establishment, 36% indicated secondary school, with 16% from college or post‐16 setting. Of the 252 respondents, 248 indicated lunch was provided in their education establishment. In the current study, an adapted socioecological model to include various levels of school food influences was used to describe and understand the views and perceptions of survey respondents (Story et al., 2009). Figure 2 displays the results as perceptions and views within the findings of the current study.

Ninety‐one per cent of respondents stated that their school food *had* changed in September 2020 (post‐lockdown), when compared to March 2020 (pre‐lockdown). Twenty‐three respondents stated no change, and of these six added comments suggesting improvements could be made to their school food provision, with regard to time, healthier or specific dietary choices and dining room arrangements.

Statements or suggestions from respondents who indicated ‘no change’ to school food provision as a result of COVID‐19 guidelines.

I don't think there has been any changes in terms of nutrition and availability, it was poor before Covid and continues to be poor after. Sadly what is served in the school canteen does not mirror the messages delivered in food lessons… ID: 6522729 (staff)

More healthy choices. Needs to be more choice for vegetarians/vegans ID: 7433009 (parent)

More gluten free options. Food available at break times. ID: 8500995 (parent)

​

Where respondents reported change, 77% indicated that lunchtime had been shortened in September 2020, with 44% reporting that school food provision was less healthy, and 50% reporting no change to the healthiness to school food provision between March and September 2020.

### Views and suggestions on school food provision post‐COVID‐19

Three main themes emerged from the overall survey data: ‘longer time to eat’, ‘reduced menu and grab items’, ‘could be healthier’, as shown in Figure 1. The results demonstrate the most important factor for all categories of respondents (student, parents and staff) was choice (Tables 2 and 3). In addition, multiple comments were made referring to specific dietary requirements not being catered for in the school provision, and sustainability with regard to increased use of plastic packaging. Table 2 demonstrates the factors deemed important, as observed from the free text responses to question 13: 'In your view how could school food experience be improved?' There was a total of 163 responses (*n* = 21 students, *n* = 107 parents, *n* = 35 staff). More choice was a common factor, holding most importance to students in this section of the survey (55%; vs. parents 31%, staff 24%). Creating more time to consume lunch was of particular importance to parents (34%) with staff showing equal concern in relation to time and choice, closely followed by ‘more healthy options’ (22%). Parents referred to their child missing lunch or swapping to home brought packed lunches often (32%). Students were concerned about time to a lesser degree, with healthier options factored as being more important (25%), followed by ‘more veggie/vegan options’ and time as equal priorities (20%).

**TABLE 2:** Question 13: Responses to ‘In your view how could school food experience be improved?’ (*n* = 163 total responses)

Emerging themes	Parents, *n* (%)	Staff, *n* (%)	Students, *n* (%)
More choice	33 (31%)	9 (24%)	11 (55%)
More time	34 (34%)	9 (24%)	4 (20%)
More healthy options	23 (22%)	8 (22%)	5 (25%)
Hot/sit‐down meal	13 (12%)	7 (19%)	0
Dining room/space	4 (4%)	5 (13%)	0
Specific dietary, vegetarian/ethnic options	5 (5%)	1 (3%)	4 (20%)
Moved to packed lunch/skipping lunch	12 (32%)	1 (3%)	1 (3%)
Cost	4 (4%)	1 (3%)	1 (3%)
Sustainability/less plastic	3 (3%)	0	1 (3%)
Positive comments	2 (2%)	1 (3%)	0

**TABLE 3:** Question 14: 'Any other comments about the changes to your school food provision?' (*n* = 112 responses)

Emerging themes	Parents, *n* (%)	Staff, *n* (%)	Students, *n* (%)
Limited choice	45 (62%)	18 (67%)	8 (67%)
Poor nutritional quality	17 (23%)	8 (30%)	3 (25%)
Hot/ sit‐down meal	14 (19%)	5 (18%)	1 (8%)
Time	0	2 (7%)	0
Changed to packed lunch/skipping lunch	13 (18%)	0	0
Positive comments	9 (12%)	2 (7%)	0

Table 3 provides the priorities as observed from question 14, eliciting free text responses: ‘Any other comments about the changes to your school food provision?’ There was a total of 112 responses (*n* = 12 students, *n* = 73 parents, *n* = 27 staff). The limited menus and choice as a result of COVID‐19 guidelines featured as the most adverse aspect of the changes to all three categories of respondents (parents 62%, staff and students 67%). Poor nutritional quality as a result of the changes to school meals was secondary to ‘choice’ for all respondents, and most frequently mentioned by staff respondents (30%) followed by students (25%) and parents (23%).

Student respondents valued choice as the most important factor, with healthy options and time as secondary factors. Interestingly, time was deemed as the most important factor by parent respondents in question 13 (Table 1), closely followed by choice, and thirdly healthy options. Staff respondents deemed choice and time of equal importance, closely followed by healthy options.

### Longer time to eat

Parents expressed concerns about time in response to the question around ‘how to improve the food experience’. Concerns about shortened lunchtimes and the impact of late lunches caused some young people to change to packed lunches brought from home or skipping lunch altogether.

My youngest has to eat her dessert first to allow her meal to cool enough, as they have only 30 minutes to queue, be served, eat, vacate. This doesn't encourage healthy options being eaten as a priority ID: 6118251 (parent)

My daughter now eats pizza at morning break because it's hot and it takes so long to get food at lunchtime by the time she's finished lunch is over… lunch is now a packet of crisps which is pretty awful… ID: 6193639 (parent)

Due to shorter lunch times ‐ child now going without school lunch ID: 6145187 (parent)

​

Of the staff who provided free text responses, time was a concern due to rushing the students to eat lunch quickly to make sure everyone eats in their respective bubbles, and that time for cleaning was factored in.

Longer periods of time to eat. Bubbles are rushed through in 5 mins and some getting pushed out after just arriving in the dinner hall. Pupils could take their lunch back to bubble locations then outside. ID: 6127271 (staff)

Lunch time has been reduced to 30 mins and only Year 7 and 6th form students are now entitled to a hot lunch…needs to be extended as students do not have time to eat, fill up water bottles and/or use the toilets… ID: 6582219 (staff)

Shorter time to each. VI formers encouraged to go off site which often means they are eating fast food and energy drinks’ ID: 6141434

​

The student respondents, in contrast to parents and staff, commented on time to a lesser degree than choice and healthiness; however, frustrations with queuing and suggestions for a longer lunchtime were expressed in the data.

Longer lunch breaks. Currently 30 mins, with 20 mins stood queueing for food ID: 6122858 (student)

Extend lunch time as queuing takes up half of the lunch time then you don't have long to eat ID: 7568429 (student)

​

### Reduced menu, grab and go items

Choice was the most prevalent theme across all three categories of respondents (parents, staff and students). Some respondents, in receipt of free school meals, suggested that the new options changing from a hot meal to only a sandwich were not filling or acceptable, perceiving a packed lunch from home would be better for the student.

Parents expressed concerns over limited choices, poor quality, convenience foods offered and less availability for those eating at the later lunchtimes. Students often chose to take in a packed lunch or skipped lunch. Suggestions for improvement included: offering more sit‐down meals at staggered lunchtimes and preordering. A few comments suggested that young people were happier with pre‐packaged fast food/HFSS options.

The choices were so poor that we moved the children onto packed lunches’ ID: 8316115 (parent)

Due to the staggered time for lunch, at least once and sometimes twice a week there is not sufficient choice for my child and she goes without any lunch! ID: 6118633 (parent)

Child will only eat pizza which is poor in terms of nutrition. Complains there is very limited menu. ID: 6217013 (parent)

​

Staff respondents demonstrated a shared priority of choice and time in their concerns arising from changes in the school food provision, with smaller portions also mentioned and one member of staff suggesting that those children living with a disability were not catered for due to the changes. Sit‐down meals were also mentioned, with some concerns raised about the difference in experience within school due to the logistics of year group bubbles, and staggered lunchtimes. For example, where student's dietary needs were not being met, or the choice/quality of lunch was dependent on the time or location students were eating their lunch.

Better quality of food needed. Meals need more flavour. Portions are too small. Not enough choice. Pupils with disabilities need a hot dessert or a soft one. ID: 6434587 (staff)

Some areas have full kitchens and can serve a full range of hot food such as chips and veg where the “pop up” kitchens have limited range ID: 7518875 (staff)

More choice for pupils who eat last…they are left with the things no‐one else wants. ID: 7456126 (staff)

Lack of choice, even poorer quality of produce which I didn't think was possible Bland taste. Food is often cold by the time pupils get it… ID: 6434587 (staff)

​

Student respondents demonstrated a higher emphasis on choice and variety through the free text responses, with four students suggesting more vegetarian/vegan or dairy free should be available. A student eligible for free school meals commented on having ‘only a sandwich’ as hot meals have been removed in the school they attend.

So the school has cut all hot meals. And it's only a sandwich as I receive free school meals I don't get as much anymore I can only get a sandwich. ID: 7703286 (student)

More choice bigger portions ID: 7898716 (student)

there could be more options for vegans and vegetarians ID: 7739163 (student)

​

### Could be healthier

Respondents commenting on nutrition or healthy options expressed a frustration with reduced nutritional quality and suggested improvements to include more fruit and vegetables and a reduction in HFSS options. Forty‐four per cent of respondents thought that school food was less healthy than pre‐COVID‐19. The percentage of respondents who requested healthier options (Table 2) was close in spread between the three respondent categories (students 25%, parents 22%, staff 22%). Parents generally agreed that all options should be healthy, also suggesting an increase in fruit and vegetables or a reduction in unhealthy choices.

Healthy food doesn't look appealing ID: 6132328 (parent)

more healthy choices. Less availability of unhealthy food as this is what they will choose everyday ID: 6289888 (parent)

My son's school offers bacon rolls or pizza as a snack at break time. These are not a snack. ID: 6304103 (parent)

Child will only eat pizza which is poor in terms of nutrition. ID: 6217013 (parent)

My son's bubble was sent home… to self‐isolate…The head teacher dropped a bag a food off which was kind but misguided. The bag contained apples and brown pitta, all good. But it also contained a 6 pack of crisps and a 5 pack of chocolate bars. I don't buy these items myself. Hardly nutritious for other families who may struggle to buy healthy food. In addition, the main food source was a box of soup sachets containing just under 100 kcals, again not sufficient as a meal. Being poor should not equal eating poor food… And school cooks also need more training. ID: 6304103 (parent)

​

Staff comments reflect their view that schools could provide healthy options, despite the COVID‐19 guidelines. The limited salad, fruit and vegetable options, and the increased fast food options were expressed by staff respondents as a concern. The range of comments of this type was from a range of geographical locations, demonstrating this is not a geographical issue. One staff respondent commented that as a food teacher they had ‘no information on canteen’.

More nutritious options. There is one main meal choice at our school now instead of three. Other hot food served is pizza, pastry products, pasta pots with limited veg and covered in cheese. Cold options consist mainly of sandwiches with a high proportion of mayonnaise and very limited salad options. ID: 6211006 (staff)

Lots of HFHS options, no fish, pizza served most days and Chips once a week. Slush puppies served…Cheap sausage and bacon sandwiches available every day. Poor quality in my opinion. ID: 6522729 (staff)

Too limited to non‐nutritious quick options like pizza slices ID: 6223679 (staff)

​

Of those students who responded to the free text questions, most noted they would like to see ‘more healthy options’.

Offer more nutritional food such as fruit or vegetables. Reward individuals for choosing healthier options within the current food selection. ID: 8554800 (student)

the vegetarian is limited and we find lunch options aren't as good or healthy ID: 6134576 (student)

In my school, there is less cold food and in the hot food section it's lukewarm instead of hot. Furthermore there is more unhealthy food at the counter which concludes in us getting a bit more fatter. ID: 6528383 (student)

​

### Schools are doing their best

Some parents and staff added comments to express their views that schools were doing their best to at least offer something within the COVID‐19 guidelines, with one parent preferring that their children like the newly introduced fast food options. There is evidence to suggest schools are varied in their approaches, and the data show good practice examples in finding other ways to keep healthful but convenient options, such as introducing a pasta bar with various toppings.

It is better than pre Covid due to staggered lunch breaks and most taking packed lunch, there's no queue ID: 6127806 (parent)

The changes have actually worked better for my fussy kids with hot sandwiches and burgers, jackets ID: 6186929 (parent)

My eldest was going into town for lunch so actually has to make healthier choices now they have to remain in school ID: 6118251 (parent)

School has just introduced last week a pasta bar so various pasta and sauces/toppings, they love it! ID: 6297408 (parent)

My daughter's school does a takeaway lunch one day a week for each year group…There is not as much choice as previously but it doesn't seem to be an issue, she still eats something. If anything it has made her try new things. ID: 7450620 (parent)

​

More participant views are displayed in Table S2.

## DISCUSSION

This study suggests that COVID‐19 has presented further challenges to improving the nutrition intake of young people in a school setting. A clear pattern, revealed in the national survey data, indicated that a significant number of secondary or post‐16 establishments may have been further from the School Food Standards (DfE, 2019) before the first school closures due to COVID‐19 (March 2020), despite government guidance stipulating schools ‘must comply with School Food Standards’ (DfE, 2020). That data suggest many schools are not serving ‘one or more portions of vegetables/salad and fruit per day’ as outlined in the School Food Standards resources (DfE, 2019). In the current study, where students are quoted as consuming ‘grab and go’ options such as a bacon bun, slice of pizza or a bag of crisps at lunch or breaktime, this evidence indicates non‐adherence to the stipulations of ‘no snacks, except nuts, seeds, vegetables and fruit with no added salt, sugar or fat (applies across the whole school day)’. The Department for Education COVID‐19 school food guidance advises ‘hot lunches should be provided wherever possible to ensure that all pupils are able to eat at least one hot meal every day’ (School Food Plan Alliance, 2020).

Evidence suggests that mal/undernourishment results in a weakened immune function, and those with obesity and co‐morbidities such as type 2 diabetes are more likely to suffer worsened outcomes from COVID‐19 (GNR, 2020). The pandemic adds more of a challenge for obesity prevention, particularly as children and young people from economically disadvantaged areas are most vulnerable (PHE, 2020; Tester et al., 2020). Current research suggests COVID‐19 has relatively mild effects on children and young people (Chanchlani et al., 2020). The indirect health implications include delayed or limited access to health care, along with the potential of increase in adverse childhood trauma (family stress, violence, poor mental health; Chanchlani et al., 2020). However, it is yet unknown how these implications may impact school food provision going forward.

### The length of school lunch: Impact on food choice and overweight

The current findings demonstrate that cleaning procedures and the logistics of keeping year groups apart as set out in the COVID‐19 guidance (DfE, 2020) have negatively impacted the time young people have to eat their lunch. However, the length of break times including lunchtimes is a pre‐existing issue, as these have reduced in the UK since 1995 (Blatchford, 2019). Blatchford (2019) examined changes to school lunchtimes in England for state‐funded secondary schools between 1995 and 2017. The study reported overall reductions in break and lunchtimes, with half of the schools having lunchtimes of <45 minutes and a quarter reported as having >35 minutes (Blatchford, 2019). Reported reasons for the time reductions prior to the COVID‐19 pandemic were related to ensuring sufficient time for learning and to limiting behavioural issues, which could take place during break/lunchtime. Although specific timings were not part of the questionnaire in the current study, some staff and young people mentioned students having only 5–10 minutes sit‐down time. Ensuring sufficient time to eat food is often discussed within a social context (Lalli, 2019). The school dining experience is an opportunity for social competence, and this learning may be fundamental to human dignity (Harrell, 2017). A longer school lunch has also been found to be a protective factor in child overweight (Bhatt, 2014), as shortened times may be more conducive to eating habits which contribute to obesity, such as eating quickly, and the consumption of foods which are convenient and energy dense (Harrell, 2017). There is limited evidence on the optimal time for school lunches, although, in a study measuring the relationship of time to food and dietary intake, young people were more likely to consume more vegetables when given at least 25 minutes of sitting time (Cohen et al., 2016). In agreement with the current findings where queuing adversely impacted young people's school food experience, Sharma et al. (2017) suggested that healthy food was more likely to be chosen when there was reduced queuing/waiting time (Sharma et al., 2017).

### It's fast and it's unhealthy

These data highlight the significant challenge the COVID‐19 guidelines have posed to effectively delivering a positive dining experience, and nutritionally balanced school food, with 44% of respondents stating the school food is less healthy than pre‐COVID‐19. The survey responses in the current study demonstrated the perceived increase in fast unhealthy food choices in schools since September 2020. The limited choice of school food options as a direct result of insufficient space or cooking areas, and a need to feed young people ‘something’ in limited time frames may have compromised the nutrient content of many school food offerings, with pizza, paninis and bacon rolls commonly featured. Parents who responded were in general agreement that all choices should be healthy, as they perceived young people would choose the unhealthy option. All respondents (regardless of category) suggested less HFSS and more fruits and vegetables should and could be available. The concern that we are moving further away from ‘the healthy choice being the easiest choice’ and the target of reducing obesity levels requires urgent attention, as eating habits developed by or during adolescence are likely to remain through adulthood (CSJ, 2017).

Adolescent eating behaviours are highly complex, and the cognitive development of food familiarity is of significant interest with regard to taste preferences towards HFSS options (Aldridge et al., 2009). Food familiarity can be encouraged through multiple tasting exposures to a variety of foods; therefore, low exposure of healthy foods from early years can affect food choices into adolescence and beyond (Aldridge et al., 2009). Furthermore, young people from less economically deprived families may be more likely to try a variety of foods (Flight et al., 2003). Contributory factors are multilevel and include the following: affordability of fruits and vegetables, fast food access, familial and community attitudes, marketing of HFSS and cultural norms (Dammann & Smith, 2009; Murphy et al., 2020).

### Skipping lunch or a home brought packed lunch…

The current data highlight young people are often missing lunch or have changed to a packed lunch prepared from home. The reasons presented in the data include smaller portions, staggered timings or waiting time increasing. The data reveal the value of having a packed lunch is also linked to the ability to snack at other times of the day and with insufficient time to consume lunch. In the past, home‐prepared packed lunches have evaluated poorly with regard to nutrition in comparison with school meals (Pearce et al., 2013). Decisions influencing the contents of the packed lunches are again multitudinous, including convenience, individual taste preference/familiarity and cost (O'Rourke et al., 2020). However, more recently Taher et al. (2020) found older adolescents consuming more nutritious home brought lunches than observed in studies of younger children. In addition, as suggested by some parent responses in the current study, where young people leave school to purchase from local food outlets, the poorest food choices are made (Taher et al., 2020). Also, adolescents missing lunch or breakfast may adversely impact dietary quality, and consuming lunch may be associated with lower intakes of sugar and total fat (Medin et al., 2019).

### Influences within a socioecological model

Using a socioecological model may support the understanding of the multiple levels of influence which impact school food choice (Story et al., 2009). Figure 2 displays the perceptions and views within the findings of the current study, contributing to the perceived barriers and potential positive actions to be taken as shown with the current data. The model draws attention to the national school food policy adherence at the macrolevel, whilst in the school environment, influential factors include food policy, dining space, time for lunch and the offer of healthy convenient options. At the level of the individual student, the importance of understanding the young person's priorities in their school food choices. These factors may support and inform effective sustainable intervention to improve school food.

A deficiency of literature examining the views and perceptions of those involved in the delivery of healthy school food promotion and provision, including school leads, catering staff and suppliers, restricts the identification of effective whole systems approaches to nutrition education and opportunities to eat healthy food in the school setting (Turunen et al., 2017). The willingness and capacity of headteachers may be a significant factor in a school's overall ‘healthy eating’ culture (Kitchen et al., 2013). Also, little is known of how schools’ contextual differences present a challenge to the development of consistent approaches, and therefore, more needs to be understood with regard to school types and demographics (*e.g*. location, food access and the number of students living with food insecurity; Tester et al., 2020). COVID‐19 has highlighted and further exacerbated problems in the school setting as a health promoting environment. Although evidence continues to emerge, currently this remains sparse. The issues in the delivery of healthy food to free school meals from the January 2021 third lockdown suggested there may be a requirement for expert nutrition advice for school catering suppliers. Although in response to poor quality food parcels being offered to free school meal pupils, the government guidance was amended. In January 2021, the option to utilise the voucher scheme to allow parents/carers to purchase food was reintroduced, along with guidance for school catering in producing food parcels, which meet the School Food Standards (DfE, 2021).

Nevertheless, this study shows that parents and students value healthy school food provision, and that many of them perceive the healthiness of food has decreased as a result of COVID‐19 guidance. As such, attention to improving school food, given the current climate and the extreme pressure schools are under, as recognised by respondents in the survey is urgently required.

### Limitations

One of the main limitations of this study is that nutritional analysis of school food has not been conducted, and therefore, findings are reliant on the self‐reported views of students, parents and staff, and their perceptions of healthiness. Due to ethics requirements and the rapid response time required to collect the data, it was only possible to obtain views from students aged 16–18 years, therefore, the data are not fully representative of the wider adolescent student experience. The survey was largely representative of the North East and predominantly consisted of White British participants; therefore, it was not possible to run inferential statistics to gage the impact of COVID‐19 guidance on different areas or ethnicity. It was therefore decided that descriptive statistics alongside the thematic analysis would befit the aim of exploring the barriers and facilitators. This also provides a limitation in the potential of transferability to all geographical areas. Also, important to note, the extent of the issues revealed in the data being solely attributed to guidance related to the current pandemic is difficult to determine without recent evaluation of school food, and indications of problems already existing.

### Recommendations for practice and policy

The good practices revealed in the data show that there are schools managing to deliver a healthy service, albeit a minority with regard to the data sample. Young people value choice, availability of healthy options and value for money. Where time is limited, efforts should be made to create convenient healthy choices and incorporate strategies to reduce waiting time, which will increase the time young people have to eat their lunch. Recommendations of good practice where possible within COVID‐19 guidance include:

- having multiple areas for serving of school lunch and where there is challenge in serving hot meals to all year groups, having alternating weeks of hot sit‐down meals and cold lunches, to ensure fairness in the range of foods served to all year groups;
- keeping all students on school premises over lunchtime and break times (although this is normal practice in many schools, the survey identifies some schools allow students off premises at lunchtime);
- offering choices which provide nutritious options (*e.g*. a pasta, rice/wrap station which has a variety of toppings);
- be smart and inclusive with food choices which meet specific dietary requirements and incorporate (*e.g*. gluten free, vegetarian/vegan). This may include overnight oat pots with a choice of toppings and rice dishes;
- nutritionists and dietitians should be consulted to provide advice on appropriate nutrition within restrictions including budget and limited preparatory equipment, in order to best support children and young people's nutritional needs.

​

Schools with in‐house catering models may benefit from external support in the form of catering staff training, local authority involvement in implementing ‘whole school’ approach, budgets and accessing a range of local suppliers. National guidance for school meals with an understanding of the current restrictions may help schools to include best practice (e.g. whole‐meal as the default option, fruit/vegetable pots). Where external catering contracts are used by schools, guidance specifies the School Food Standards should be included within the catering contract or service level agreement and stipulates regular evidence of compliance to be supplied to the school's governing body (DfE, 2020).

In addition, it may be useful at national level to supply schools with resources for the educating of young people on the importance of good immune health. Future research to examine the nutritional content of school food provision, and adherence to national school food policy may provide further insight into the effectiveness of school food provision in the UK.

## CONFLICT OF INTEREST

None.

## ETHICAL APPROVAL

This study was conducted according to the guidelines laid down in the Declaration of Helsinki, and all procedures involving research study participants were approved by the School of Health and Life Sciences research ethics sub‐committee at Teesside University (Ref: 2020 Sep 1325 Rose). Written informed consent was obtained from all subjects.

## Supporting information

Tables S1‐S2
