# Supplementary material for: ‘Pizza every day – why?’: A survey to evaluate the impact of COVID‐19 guidelines on secondary school food provision in the UK
Source: Nutr Bull. 2021 Jun 4;46(2):160–71. doi: 10.1111/nbu.12496 (PMC8206956; doi:10.1111/nbu.12496)
Supplement: Supplementary file 1 — Tables S1‐S2 [file NBU-46-160-s001.docx]

Supplementary (S) Table 1: Copy of survey questions

| Please confirm you are 16 years or over |
| --- |
| **Please type the first three characters of your postcode** |
| **Tick one of the below as appropriate:**  Parent Staff student |
| **Year group of you/ your child/ group you teach/serve (tick all that apply for school staff serving/teaching)**  7 8 9 10 11 12 13 |
| **What is your ethnic group?**  Choose one option that best describes your ethnic group or background  **White**  1. English/Welsh/Scottish/Northern Irish/British 2. Irish 3. Gypsy or Irish Traveller 4. Any other White background, please describe  **Mixed/Multiple ethnic groups**  5. White and Black Caribbean 6. White and Black African 7. White and Asian 8. Any other Mixed/Multiple ethnic background, please describe  **Asian/Asian British**  9. Indian 10. Pakistani 11. Bangladeshi 12. Chinese 13. Any other Asian background, please describe  **Black/ African/Caribbean/Black British**  14. African 15. Caribbean 16. Any other Black/African/Caribbean background, please describe  **Other ethnic group** |
| **Please select type of education establishment you attend/ your child attends/ you work at**  Post 16 College Secondary academy Secondary school |
| **Is school lunch provided in your/your child’s school?**  Yes No  If **Yes** please answer the following questions, if **No** thank you for your participation, you are not required to complete the following questions. |
| **Has the school food choice changed from September 2020 compared with March 2020? (e.g. limited range/ cold food only)**  If **Yes** please answer the following questions, if **No** thank you for your participation, you do not need to complete the following questions. |
| **Please tick the relevant box to indicate if school food options are currently hot only/cold only or both:**  Hot Cold Both |
| **Do you think the choice is less or more healthy/nutritious than before COVID-19?**  Less same More |
| **Has the time allowed for school lunch to be eaten changed since September 2020 compared with March? Please tick the relevant box:**  shorter longer same/no change |
| **In your view how could your school food experience be improved?** |
| **Please add any relevant information about the food choices available now in your school, and your views on the impact of the new COVID-19 school guidelines on your school food.** |

Supplementary Table 2: STROBE checklist

|  | Item No | Recommendation | Page  No |
| --- | --- | --- | --- |
| **Title and abstract** | 1 | (*a*) Indicate the study’s design with a commonly used term in the title or the abstract | 1 |
|  |  | (*b*) Provide in the abstract an informative and balanced summary of what was done and what was found | 2-3 |
| Introduction | | | |
| Background/rationale | 2 | Explain the scientific background and rationale for the investigation being reported | 3-6 |
| Objectives | 3 | State specific objectives, including any prespecified hypotheses | 6 |
| Methods | | | |
| Study design | 4 | Present key elements of study design early in the paper | 6 |
| Setting | 5 | Describe the setting, locations, and relevant dates, including periods of recruitment, exposure, follow-up, and data collection | 6-7 |
| Participants | 6 | (*a*) *Cohort study*—Give the eligibility criteria, and the sources and methods of selection of participants. Describe methods of follow-up  *Case-control study*—Give the eligibility criteria, and the sources and methods of case ascertainment and control selection. Give the rationale for the choice of cases and controls  *Cross-sectional study*—Give the eligibility criteria, and the sources and methods of selection of participants | 6-8 |
|  |  | (*b*) *Cohort study*—For matched studies, give matching criteria and number of exposed and unexposed  *Case-control study*—For matched studies, give matching criteria and the number of controls per case | N/A |
| Variables | 7 | Clearly define all outcomes, exposures, predictors, potential confounders, and effect modifiers. Give diagnostic criteria, if applicable | 6-7 and 25 |
| Data sources/ measurement | 8* | For each variable of interest, give sources of data and details of methods of assessment (measurement). Describe comparability of assessment methods if there is more than one group | *7* |
| Bias | 9 | Describe any efforts to address potential sources of bias | 6-7 and 25 |
| Study size | 10 | Explain how the study size was arrived at | N/A |
| Quantitative variables | 11 | Explain how quantitative variables were handled in the analyses. If applicable, describe which groupings were chosen and why | N/A |
| Statistical methods | 12 | (*a*) Describe all statistical methods, including those used to control for confounding | 6-7 |
|  |  | (*b*) Describe any methods used to examine subgroups and interactions | 8 |
|  |  | (*c*) Explain how missing data were addressed | 25 |
|  |  | (*d*) *Cohort study*—If applicable, explain how loss to follow-up was addressed  *Case-control study*—If applicable, explain how matching of cases and controls was addressed  *Cross-sectional study*—If applicable, describe analytical methods taking account of sampling strategy | 6-7 |
|  |  | (*e*) Describe any sensitivity analyses | N/A |

| Parents  Supplementary Table 3: Quotes from survey participant data | Staff | Students |
| --- | --- | --- |
| ‘my year 7 son does not have enough time to queue up to buy his lunch and then eat it in the 30 minutes provided. he is quite a slow eater and gets distracted. he often brings his lunch home and eats it at 3.30’ ID: 6113387  ‘My youngest has to eat her dessert first to allow her meal to cool enough, as they have only 30 minutes to queue, be served, eat, vacate. This doesn’t encourage healthy options being eaten as a priority’ ID: 6118251  ‘My daughter now eats pizza at morning break because it’s hot and it takes so long to get food at lunchtime by the time she’s finished lunch is over ... lunch is now a packet of crisps which is pretty awful . She also turned vegetarian over lockdown there is now only 1 veggie option and it’s not advertised (I guess due to low numbers) ...being socially awkward and with busy canteen staff she rarely asks...’ ID: 6193639  ‘The key thing to change is the time allowed for students to have lunch. It is too short by the time they have queued and get to eat’ ID: 8306613  ‘Their lunchtime has moved to much later in the day so they often eat from their lunch between classes to sustain them.’ ID :8316115  ‘Due to shorter lunch times - child now going without school lunch’ ID: 6145187  ‘My children both take a packed lunch to school as the time they have now is very limited.’ ID: 6143888  ‘The choices were so poor that we moved the children onto packed lunches’ ID:8316115  ‘They often don’t get a lunch as often the sandwich is incorrect from the choice selected’ ID: 6124796  ‘Due to the staggered time for lunch, at least once and sometimes twice a week there is not sufficient choice for my child and she goes without any lunch! So there is a requirement to make sure food preparation is taking into account the staggered times and that not all the salad / sandwich provision is put out at once.’ ID: 6118633  ‘Not enjoying it as much - limited choice’ ID: 6119637  ‘The food choices are poor. My daughter refuses to have it and takes packed lunch everyday’ ID: 6124931  ‘More choice! It’s pasta or a sandwich. Yesterday my son had a scone for lunch!’ ID: 6152415  ‘My son's school offers bacon rolls or pizza as a snack at break time. These are not a snack. They offer pudding every day. They also don't supervise the children to see if they actually eat their meals. My son has said that the quality and variety of food has declined since they returned to school post-lockdown. He says the food is often lukewarm or seems stale...I'm assuming they keep food warm in a hotbox to cope with the longer, staggered lunch times. He often comes home hungry. He receives free school meals but I think I will have to start sending him with a packed lunch otherwise he often returns home ravenous’. ID: 6304103  ‘At my daughter’s school there doesn't seem to be a varied choice and the food tends to be same everyday. They are currently only having quick sandwiches, pasta pots, burgers etc and not been offered a full meal as such.’ ID: 7723164  ‘Greater selection of cold food / sandwiches / salad if these are wanted’ ID: 6299198  ‘Pasta king one week and hot the other. Poor choices for food and no higher quality’ ID: 6124931  ‘Menus no longer available. My daughter tells me lunches are pre packed with sandwich, drink, cookie. No options possible’ ID: 6190290  ‘Child will only eat pizza which is poor in terms of nutrition. Complains there is very limited menu.’ ID: 6217013  ‘Pizza and panini choices with chicken and chips on a Friday’ ID: 6283257  ‘Seems there are more quick choices rather than meals to sit down and eat’ ID: 6288215  ‘My sons school had a wide range of options before covid but has a smaller selection now. The very unhealthy options shouldn’t be available at all in my opinion and this is what he will choose’ ID: 6289888  ‘Pizza every day - why? hot choice is reduced, pre packed sandwiches with lots of salt not very healthy. remove energy drinks’ ID: 7158291  ‘My child doesn’t get a choice of a hot meal and cold option is very limited because of set lunch times now in force and younger year groups going first (very little food then left for years 10 and 11).’ ID: 6113210  ‘The choices differ depending on which outlet they are collecting from - they change every week - some weeks my children are hungrier than others as they don’t really like what’s on offer at that outlet that week! They would both prefer packed lunches at the moment!’ ID: 6120073  ‘My children haven't complained about there being less choice however snack choices available are high in sugars e.g. doughnuts, nutrigrain bars, biscuits, cakes.’ ID: 6116405  ‘Students have to have 1 veg with hot meal but as the Carrots are undercooked, broccoli like mash, & the mash lumpy, child often bins it 😕. (Child eats veg at home!) quality affects what is eaten.’ ID: 6140633  ‘The changes have actually worked better for my fussy kids with hot sandwiches and burgers, jackets’ ID: 6186929  ‘Healthy options to be reinstated across all year groups’ ID: 6122495  ‘Healthier options, no fizzy drinks or chips served would be a good start’ ID: 6130802  ‘lunch is now a packet of crisps which is pretty awful’ ID: 6193639  ‘By offering only healthy food at the café/canteen’ ID: 6195928  ‘Healthy food doesn’t look appealing’ ID: 6132328  ‘not serving chips or cookies but focusing on healthier options’ ID: 6228697  ‘Snacks should be healthy no unhealthy snacks and fresh fruit available’ ID: 6274668  ‘more healthy choices. Less availability of unhealthy food as this is what they will choose everyday’ ID: 6289888  ‘Only healthy options available – as its self choice we don’t have much say in what they choose’ ID: 6311080  ‘More fresh fruit and veg available at break times’ ID: 6347786  ‘My son's school offers bacon rolls or pizza as a snack at break time. These are not a snack.’ ID: 6304103  ‘snack choices available are high in sugars e.g. doughnuts, nutrigrain bars, biscuits, cakes.’ ID: 6116405  ‘Morning break offerings could be healthier (pizza at 10am!?)’ ID 6194343  Very limited and nutritionally lacking! ID: 6152415  ‘I do not feel they are receiving a wide enough range of healthy food to provide them with a varied balanced diet.’ ID: 6153562  ‘Only junk/unhealthy food so my children do not eat there’ ID: 6195928  ‘Child will only eat pizza which is poor in terms of nutrition. Complains there is very limited menu.’ ID: 6217013  ‘My sons tend to gravitate towards pasta king most days. I would like to see this containing more additional nutritious ingredients’ ID: 6288180  ‘The very unhealthy options shouldn’t be available at all in my opinion and this is what he will choose.’ ID: 6289888  ‘My son's bubble was sent home… to self isolate…The head teacher dropped a bag a food off which was kind but misguided. The bag contained apples and brown pitta, all good. But it also contained a 6 pack of crisps and a 5 pack of chocolate bars. I don't buy these items myself. Hardly nutritious for other families who may struggle to buy healthy food. In addition, the main food source was a box of soup sachets containing just under 100kcals, again not sufficient as a meal. Being poor should not equal eating poor food, while it was kind of the head teacher he could have sought advice on how to purchase healthier options with the allocated budget. Perhaps focus on fibre and protein rather than sat fat and sugar. I ended up having mini battles with my son over the crisps and biscuits. Being at home for ten days made physical activity challenging, there was no way I was going to let him eat rubbish too. I think educators are really quite uninformed when it comes to nutrition. And school cooks also need more training.’ ID: 6304103  ‘Pizza every day - why? hot choice is reduced, pre packed sandwiches with lots of salt not very healthy. remove energy drinks’ ID: 7158291  ‘The current offer includes Burritos cheese burger, chicken wraps hot dogs, pasta pots, sandwiches. I would prefer to see healthier options and ideally a hot meal option. Fruit bowls or pre prepared healthy lunch boxes do that it’s still COVID safe.’ ID: 8518039  ‘I think it is better than it was, no long waiting times for lunches now.’ ID: 7155618  ‘Think the school has done everything they can to provide children with a good meal either hot or cold. I’m a parent but also work in the catering department of the school they attend’ ID: 7457100  ‘My eldest was going into town for lunch so actually has to make healthier choices now they have to remain in school’ ID: 6118251  ‘He would happily eat most things on the menu from the good selection the school Offers.’ ID: 6125776  ‘My son has commented on how the options have improved, offering a much wider menu’ ID: 6153129  School has just introduced last week a pasta bar so various pasta and sauces/toppings, they love it! ID: 6297408 | ‘Longer periods of time to eat. Bubbles are rushed through in 5 mins and some getting pushed out after just arriving in the dinner hall. Pupils could take their lunch back to bubble locations then outside.’ ID: 6127271  ‘Longer to eat their lunch - we are having to remove pupils so the next year group bubble can come in after it has been cleaned down’. ID: 6156016  ‘Lunch time has been reduced to 30 mins and only Year 7 and 6th form students are now entitled to a hot lunch. PP students are provided with a cold packed lunch and all other students need to bring food in from home. Lunch time needs to be extended as students do not have time to eat, fill up water bottles and/or use the toilets. Other year groups should be catered for in some capacity e.g. opening up a pre-order online system and having food delivered to form rooms for consumption’ ID: 6582219  ‘Shorter time to each. VI formers encouraged to go off site which often means they are eating fast food and energy drinks’ ID: 6141434  ‘Better quality of food needed. Meals need more flavour. Portions are too small. Not enough choice. Pupils with disabilities need a hot dessert or a soft one. ID: 6434587  ‘Wider choice as v limited now, portions are smaller too but no idea why’ ID: 6585877  ‘More choice for pupils who eat last...they are left with the things noone else wants.’ ID: 7456126  I think they do the best job with what they have however due to running more serving areas some children aren't getting the same as others and feel this is unfair ID: 7518875  Lack of choice, even poorer quality of produce which I didn’t think was possible Bland taste. Food is often cold by the time pupils get it. Have to choose at breakfast time when they are full. The choice is one hot meal, sandwich or jacket. No salad, no wraps, no option to change mind. The dessert is sometimes a biscuit which many students can’t swallow. ID: 6434587  ‘Sandwiches and a few salads appear to be the only really choice now there is no hot meal available (due to staggering lunches/ time/ Covid restrictions). This is very limited especially for children who are not fond of the choices or not being provided with healthy or sometimes even hot meals at home.’ ID: 6270889  ‘Fewer choices and also as there have had to be several dining rooms set up this has meant that food needs to be served from food trolleys so this is a great limiter’ ID: 7453535  ‘Reduced menu. Grab and go items.’ ID: 6597242  ‘The need to separate year groups means there are no catering facilities to provide hot food for 2 year groups. The choice of hot food is massively limited due to social distancing rules for catering staff.’ ID: 6904083  ‘Healthier food selection with more exposure to plant based meals and a reduction in red meat.’ ID: 6194575  ‘More nutritious options. There is one main meal choice at our school now instead of three. Other hot food served is pizza, pastry products, pasta pots with limited veg and covered in cheese. Cold options consist mainly of sandwiches with a high proportion of mayonnaise and very limited salad options.’ ID: 6211006  ‘More healthy but quick options ensuring a nutritious and filling meal’ ID: 6223679  ‘more healthier options could be as little as fruits alternatives instead of sweet snacks’ ID: 6467400  ‘Lots of HFHS options, no fish, pizza served most days and Chips once a week. Slush puppies served. Salad options are available on request but I think as these are not ‘seen’ like the hot food options students are less likely to purchase. Cheap sausage and bacon sandwiches available every day. Poor quality in my opinion. ID: 6522729  ‘Healthier choices. Fruit instead of cakes. No added sugar drinks’ ID: 6593966  ‘Too limited to non nutritious quick options like pizza slices’ ID: 6223679  ‘I don’t think there has been any changes in terms of nutrition and availability, it was poor before Covid and continues to be poor after. Sadly what is served in the school canteen does not mirror the messages delivered in food lessons and pshe.’ ID: 6522729  ‘most food options are purchased from outside sources now and no salad bar or fresh pasta service available’ ID 6578768  ‘The range of lighter/healthier options has been stopped eg the salad, baked potatoes and soup bars’ ID: 6582219  ‘More sandwiches and baguettes and cakes. Nothing nutritious’ ID: 6593966  ‘There are less salad options. Prepacked lunches were high in sugars and fairly low in nutrition as they contained a dessert item, biscuit, crisps, plain sandwich and option to add fruit items and fruit juice’ ID: 6949112  ‘it is already providing a range of good quality hot home cooked meals. Maybe slightly less English traditional and more variety on the International cuisine would be great’ ID: 6831518  ‘Not possible in current climate due to space and time limitations’ ID: 7453535  More restricted menu in place due to controls needed. However, I think it is sensible considering the balance needed between providing food and the need to ensure safety of all. All students can still access some form of hot option and a range of cold. ID: 6148228 | ‘longer lunch breaks. Currently 30 mins, with 20 mins stood queueing for food’ ID: 6122858  ‘Extend lunch time as queuing takes up half of the lunch time then you don’t have long to eat’ ID: 7568429  ‘So we had a 45 minute lunch and now has been shortened to 25 so I think we need longer.’ID: 7703286  ‘So the school has cut all hot meals. And it’s only a sandwich as I receive free school meals I don’t get as much anymore I can only get a sandwich.’ ID: 7703286  ‘More choice bigger portions’ ID: 7898716  ‘more options, salad bar to be open again , more pasta sauce choices (as there was previous to covid-19)’ ID: 8554809  ‘More selection of foods’ ID: 7776557  ‘there could be more options for vegans and vegetarians’ ID: 7739163  There is more variety as the grab and go option such as paninis and toasties. However there is less cold lunches available like less sandwiches/ salad/ wrap variety. ID: 7566089  ‘more options , salad bar to be open again , more pasta sauce choices (as there was previous to covid-19)’ ID: 8554809  ‘Offer more nutritional food such as fruit or vegetables.Reward individuals for choosing healthier options within the current food selection.’ ID: 8554800  ‘the vegetarian is limited and we find lunch options aren’t as good or healthy’ ID: 6134576  ‘In my school, there is less cold food and in the hot food section it's lukewarm instead of hot. Furthermore there is more unhealthy food at the counter which concludes in us getting a bit more fatter.’ ID: 6528383  ‘There appears to be a wider range of choice in food but not exactly a wider selection of more nutritional food. New options include stuff like different puddings and foods that wouldn’t exactly be classed as healthy.’ ID: 8554800 |
